# Three-dimensional foot shape analysis in children: a pilot analysis using three-dimensional shape descriptors

**DOI:** 10.1186/s13047-020-0373-7

**Published:** 2020-01-30

**Authors:** Matyas Varga, Carina Price, Stewart C. Morrison

**Affiliations:** 1 School of Health Sciences, University of Brighton, Brighton, UK; 2 School of Health and Society, University of Salford, Salford, UK

**Keywords:** Paediatric foot, Foot morphology, 3D scanning, Shape-index, Curvedness, Foot development

## Abstract

**Background:**

Existing clinical measures to describe foot morphology are limited in that they are commonly two-dimensional, low in resolution and accuracy, and do not accurately represent the multi-planar and complex changes during development across childhood. Using three-dimensional (3D) scanner technology provides the opportunity to understand more about morphological changes throughout childhood with higher resolution and potentially more relevant 3D shape measures. This is important to advance the prevailing arguments about the typical development of children’s feet and inform the development of appropriate clinical measures. 3D shape descriptors derived from 3D scanning can be used to quantify changes in shape at each point of the 3D surface. The aim of this study was to determine whether 3D shape descriptors derived from 3D scanning data can identify differences in foot morphology between children of different ages.

**Methods:**

Fifteen children were recruited from three age groups (2, 5, and 7 years of age). Both feet were scanned in bipedal stance, using the Artec Eva (Artec Group, Luxembourg, Luxembourg) hand-held scanner. Three dimensional shape descriptors were extracted from the 3D scans of the right foot, to create histograms for each age group and heat maps of representative participants for comparison.

**Results:**

There were changes to the dorsal, medial and lateral surfaces of the feet with age. The surfaces became less round along with an increase in indented areas. This is supported by the heat maps which demonstrated that the surfaces of the anatomical landmarks (e.g. the malleoli and navicular tuberosity) became more rounded and protruding, with indented surfaces appearing around these landmarks. On the plantar surface, the concavity of the midfoot was evident and this concavity extended into the midfoot from the medial aspect as age increased.

**Conclusions:**

The findings of this study indicated that with increasing age the foot becomes thinner in 3D, with bony architecture emerging, and the medial longitudinal arch (MLA) increases in area and concavity. Three-dimensional shape descriptors have shown good potential for locating and quantifying changes in foot structure across childhood. Three-dimensional shape descriptor data will be beneficial for understanding more about foot development and quantifying changes over time.

## Background

Providing robust data which characterises the development of the paediatric foot is needed in order to help clinicians and researchers understand the typical trajectory of the foot throughout the complex and variable stages of growth and development. Data which describes the linear anthropometry of the foot throughout childhood has existed for many years [1–5], but studies reporting anthropometric measures to describe changes in foot shape are limited in what they tell us about three-dimensional shape and development. More recent studies have adopted static clinical measures [6–9], filming [10] and pressure technology [11] to capture different aspects of foot development but there is no universal agreement on the appropriate measures for determining foot shape (or structure) [12], although there are concerns about the validity of footprint measures [6]. Equally, attempts to describe the typical development of foot shape across childhood using clinical measures such as the Foot Posture Index [6] are limited in that existing measures have not been validated in children, and are based on underlying clinical assumptions about foot characteristics.

Considering the limitations of clinical measures to accurately describe the development of foot shape across childhood, three-dimensional scanning technology shows promise. Research using a 3D stand-in scanner captured variation of children’s feet and resulted in the description of different morphological foot types [13]. Earlier work by the same authors looked at foot morphology of normal, underweight and overweight children [14] demonstrating some real-world clinical impact of this technology, although still limited to 2D measures. Understanding more about the three-dimensional shape and morphological changes through childhood is important to advance the prevailing arguments about the typical development of children’s feet [15] and inform the development of appropriate clinical measures.

Within the existing research in other fields, three-dimensional shape descriptors have already shown capacity to characterise the shape of different body surfaces [16–21] and bones [22]. This analytical approach produces shape index (a number between − 1 and 1 representing 9 distinct 3D shapes and transitions between shapes) and curvedness for each point (vertex) on the surface of the scanned object, describing the 3D shape and the surface curvature [23, 24]. Despite the application to other areas of the body, there is sparse evidence of advances with this analysis in research relating to the growth and development of the paediatric foot.

Liu, Kim [25] argued that shape-index can capture the local shape, and provide a map of convex (protruding) and concave (indented) areas which will help with the automatic localization of anatomical landmarks of the foot and lower leg. In addition to isolation of anatomical landmarks in adult feet [25], shape index and curvedness can also be used to identify shape changes due to development of the paediatric foot. As the shape and curvedness are represented numerically for each point on the surface, these can be subject to statistical analysis, hence they can be used to quantify the differences or changes in the 3D shape of the paediatric foot surface. Therefore, the aim of this study was to investigate whether 3D shape descriptors derived from 3D scanning data can help identify differences in 3D foot shape between children of different age groups. This study was designed to provide an understanding of how these measures can relate to paediatric foot development and therefore to indicate how the measures can be used in future work with larger cohorts of children.

## Methods

### Participants

In order to explore the application of 3D shape descriptors in the quantification of foot shape, a convenience sample of children were recruited from three age groups (2, 5, and 7 years of age) from local community groups and assigned to Group 1 (2 years old, *n* = 5), Group 2 (5 years old, *n* = 5) and Group 3 (7 years old, *n* = 5).

Children recruited into the study were typically developing and free from any skin disorders affecting the feet (such as eczema, psoriasis or any skin abrasions) or any diagnosis of epilepsy or light sensitive conditions.

### Data collection

**Fig. 1:**
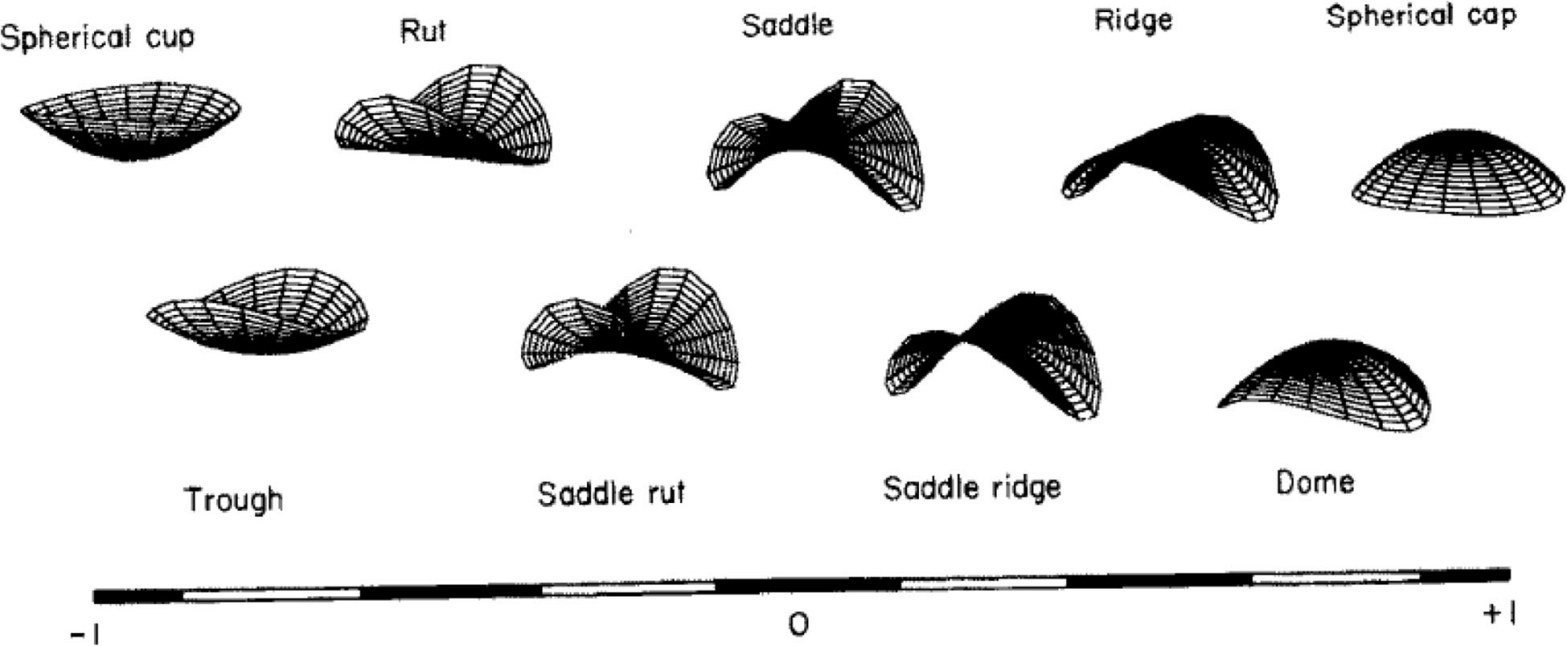
Illustration of the nine categories of shape index with detailed ranges defined in Table 1. Reprinted with permission from Koenderink and van Doorn [29]

Data collection was undertaken in the local community where a scanning platform was established. This was comprised of a custom-built stand and Perspex platform to enable scanning of the plantar surface (see Fig. 1). Scanning of the children’s feet was undertaken using the Artec Eva scanner (Artec Group, Luxembourg, Luxembourg). This is a structured light scanner and was chosen because of its superior data acquisition speed, accuracy, and resolution (acquisition speed: 2mln points/second resolution of 0.5 mm and an accuracy of 0.1 mm,). The portability of the hand-held scanner was also an important factor as this supported data capture in more typical environments that children would be comfortable with (e.g. at school, or sports club). Participants stood barefoot in a bipedal stance on the platform for approximately 120 s while the researcher (MV) scanned their feet. Scanning was performed in three separate scans. The dorsal and posterior parts of the feet were scanned first, with the researcher moving around the participant’s feet with one continuous scan. The second and third scans captured the plantar surface of the feet by moving the scanner from the lateral aspect of the foot and under the platform from either side. Participants were instructed to stand as still as possible and were encouraged to watch a video projected on the wall to distract them. Three scans of each participant’s feet were undertaken and if participants moved during scanning, it was repeated.

### Data processing

Following data capture, Artec Studio 12 software (Artec Group, Luxembourg, Luxembourg) was used for post-processing. This involved: (1) simplification: reduction of points in the models to match point numbers within age groups (2); isotropic remeshing: modifying the 3D model to make triangle sizes uniform, (3) smoothing: flattening out noisy areas. The scan for each participant was used to create a full 3D model of each foot. The models were then exported as stereolithography (stl) files to Matlab R2018a (The MathWorks, Inc., Natick, Massachusetts, United States), resulting in a point cloud for each foot. Only the right feet were used for analysis to ensure statistical independence within the samples [26]. Processing of the point clouds included computing 3D shape descriptors and comparing these between age groups for four foot surfaces: plantar, dorsal, medial and lateral. These surfaces were defined in a custom written Matlab script using the orientation of normal vectors of vertices.

### 3D shape descriptor calculation

A triangulation of the foot point clouds was performed to calculate principal and Gaussian curvature, using an amended script by Peyre [27], based on Cohen-Steiner and Morvan [28]. Using Gaussian-curvature, shape-index and curvedness were calculated according to the equation by Koenderink and van Doorn [24] in a custom written code. Curvedness (CU) ranges from zero (flat surface) to infinity. Shape-index (SI) is defined as a range from − 1 to 1, with nine different shapes defined (Table 1). Example images for the shapes described in the table can be seen in Fig. 1. The authors assessed the reliability of capturing these measures in the same sample [30] and found that root mean square error (RMSE) for 3D shape descriptors for 3 scans of the same foot decreased with age. This analysis indicated good reliability (RMSE for the 7 years old group: SI: 0.064, CU: 0.001; 5 years old group: SI: 0.071, CU: 0.001; 2 years old group: SI: 0.091, CU: 0.002).

**Table 1:** Shape-index ranges for each shape for 3D shape descriptors [24]

Shape	Shape-index range
Spherical cup	-1 to -0.875
Trough	-0.875 to -0.625
Rut	-0.625 to -0.375
Saddle rut	-0.375 to -0.125
Saddle	-0.125 to 0.125
Saddle ridge	0.125 to 0.375
Ridge	0.375 to 0.625
Dome	0.625 to 0.875
Spherical cap	0.875 to 1

### Data analysis

To compare the surfaces between age groups, their distribution of shape-index and curvedness were used. Relative probability histograms visualised the differences between age groups distribution in both shape-index and curvedness for each surface of the foot. Relative probability histograms were chosen to compensate for the differences in the number vertices between age groups influencing the results. When considering the shape-index, each bar of the relative probability histogram represented the shape defined for that range in Table 1. Shapes below zero were considered concave, and shapes over one were considered convex. Visual representation of example curvedness and shape index heat maps of surfaces from one representative participant from each of the three age groups was also included. The heat maps show the vertices in the foot scan with colours representing either curvedness or shape-index values provided on the colour bar. The heat maps represent the full foot shape (in black) and is presented for reference purposes. When comparing the plantar surfaces - because flat is not defined with a certain shape-index value - only the vertices with a curvedness value higher than 0.0015 were considered in the histograms and heat maps of the shape-index data. This was to remove flat sections of the foot and therefore based on the most common probability in the curvedness histogram.

## Results

Five children were recruited from each of the age groups: two (3 males and 2 females), five (3 females and 2 males) and seven (3 females and 2 males) years of age. The results are presented with reference to the Groups of increasing age: Group 1: 2 years old, Group 2: 5 years old and Group 3: 7 years old.

### Dorsal surface

**Fig. 2:**
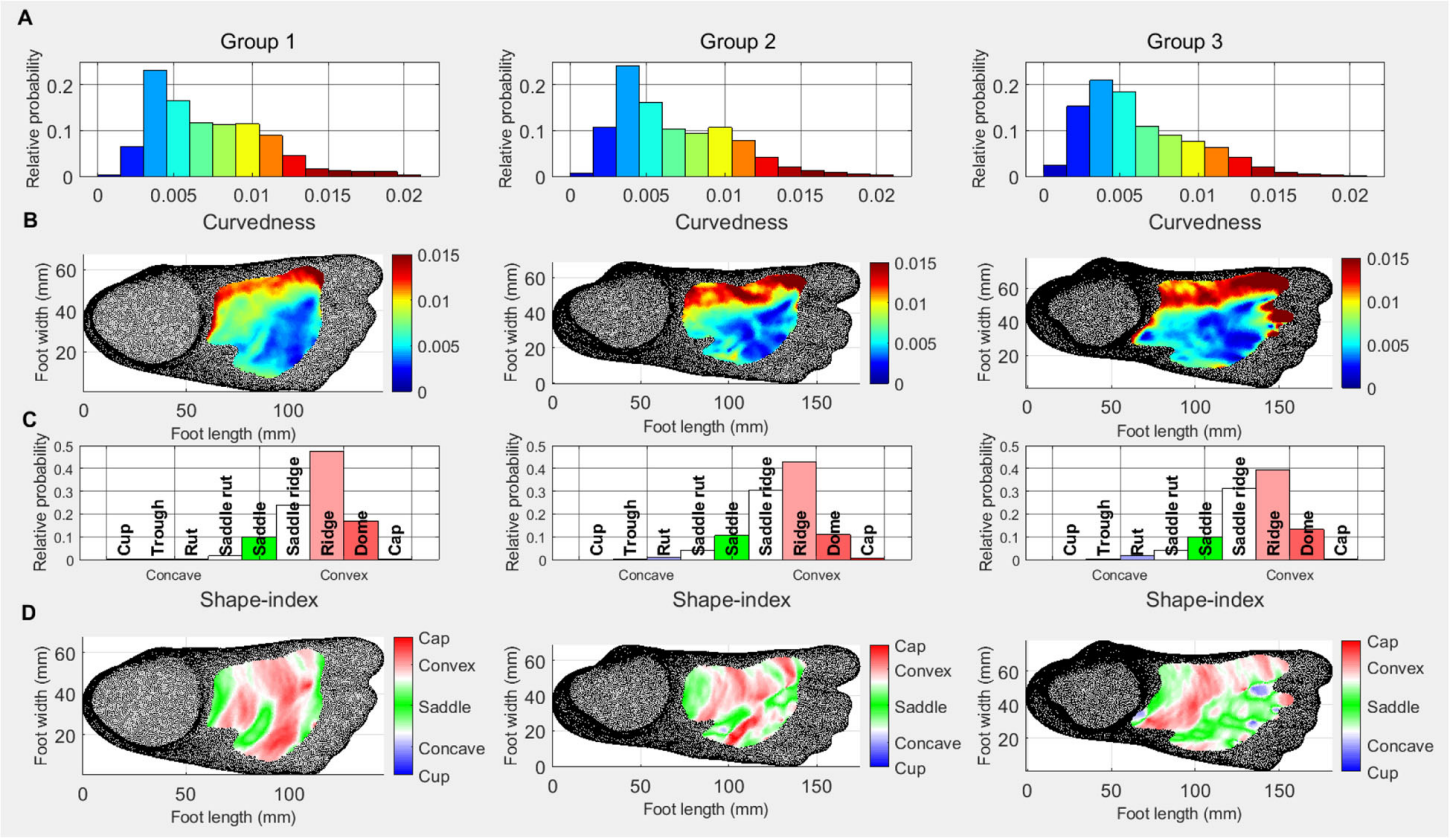
Dorsal surface: Relative probability histograms: **a** curvedness **c** shape-index; Heat maps: **b** curvedness **d** shape-index

The curvedness histograms (Fig. 2a) for the dorsal surface of the foot show an increase in the lower curvedness values and a decrease in the higher values from Group 1 to Group 2 and 3. The second peak around 0.01 decreases from Group 1 to Group 2 and disappears in Group 3.The curvedness heat maps (Fig. 2b), show the increased area of lower curvedness (as identified by the dark blue) and a decrease in medium curvedness (light green) on the lateral side of the dorsal surface with age, which agrees with the histogram and an increasing area of higher curvedness on the medial side.

The shape-index histograms (Fig. 2c) indicate an increase in concave shapes, a decrease in the ridge shape and an increase in the saddle ridge shape from Group 1 to Group 2.

The shape-index heat maps show that on the medial side of the dorsal surface, an alternating saddle-ridge - ridge pattern is developing with age. There is also a reduction of convex and an increase in saddle shape on the rest of the dorsal surface.

### Medial surface

**Fig. 3:**
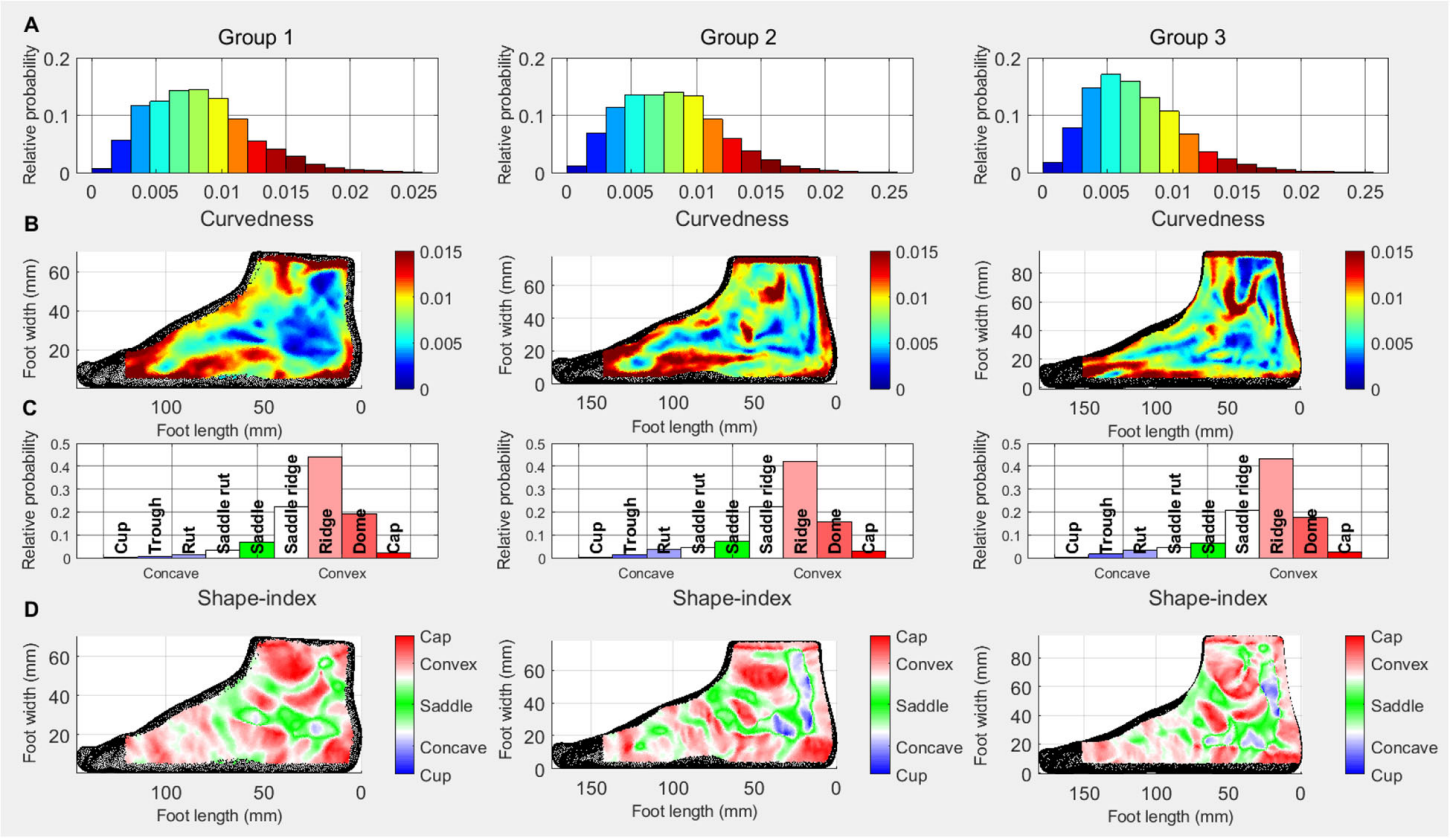
Medial surface: Relative probability histograms: **a** curvedness **c** shape-index; Heat maps: **b** curvedness **d** shape-index

Similarly to the dorsal surface, the medial surface also shows a reduction in higher curvedness values, and an increase in lower curvedness values as age increases. Figure 3b shows a decreased curvedness on the inferior medial surface in the Group 3 participant and increased curvedness around anatomical landmarks like the medial malleolus and the navicular in the Group 2 and Group 3 participants. The maps also demonstrate that curvedness decreases with age on the superior boundary of the medial surface.

The shape-index data reveals an increase in concave shapes and a slight decrease in dome shape (Fig. 3c) from Group 1 to Group 2. The shape-index heat maps show the medial malleolus, the navicular and the first metatarsal head, which are more prominent in the older children, along with concavity appearing posterior to the medial malleolus. Consistent with the pattern on the superior surface, an alternating red, white, green pattern is appearing on the superior boundary of the medial surface.

### Lateral surface

**Fig. 4:**
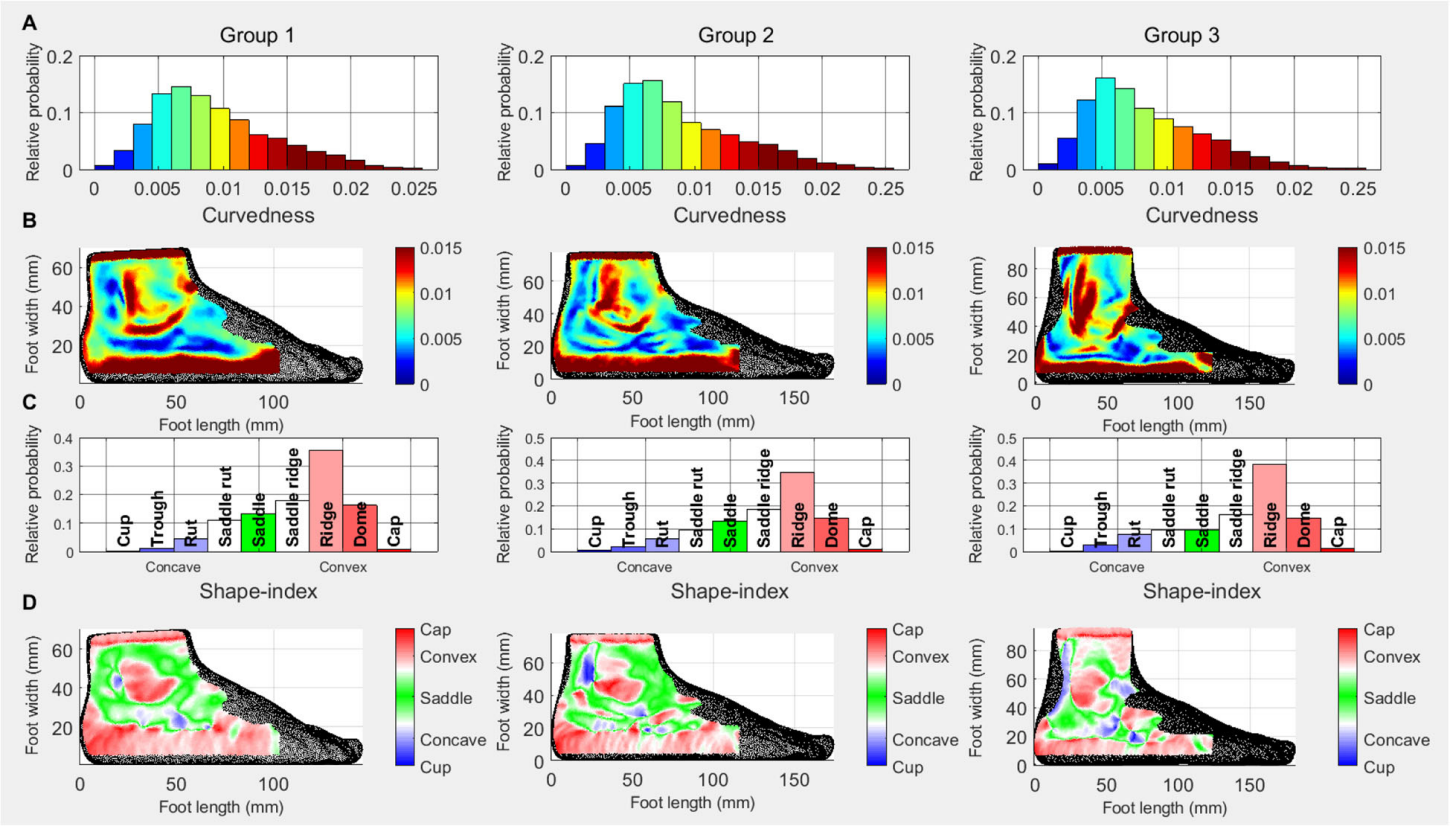
Lateral surface: Relative probability histograms: **a** curvedness **c** shape-index; Heat maps: **b** curvedness **d** shape-index

The curvedness histograms (Fig. 4a) of the lateral surfaces demonstrate a shift towards lower curvedness values with increasing age. The curvedness heat maps (Fig. 4b) show a shift in the curvedness of the lateral border of the foot and the increase in size and curvedness of the lateral malleolus with age. A higher curved structure inferior and anterior to the lateral malleolus is apparent in the Group 1 and Group 2 participants which disappears or shifts more anteriorly in the Group 3 participants.

The main differences between the Groups in shape-index are the increase in ridge shape (Fig. 4c) and the reduction in saddle shape in Group 3 and an increase in concave shapes as age increases. The shape-index heat maps demonstrate the development of the lateral malleolus (see Fig. 4d). The concave shape-index values for Groups 2 and 3, mainly appear posterior and anterior to the lateral malleolus.

### Plantar surface

**Fig. 5:**
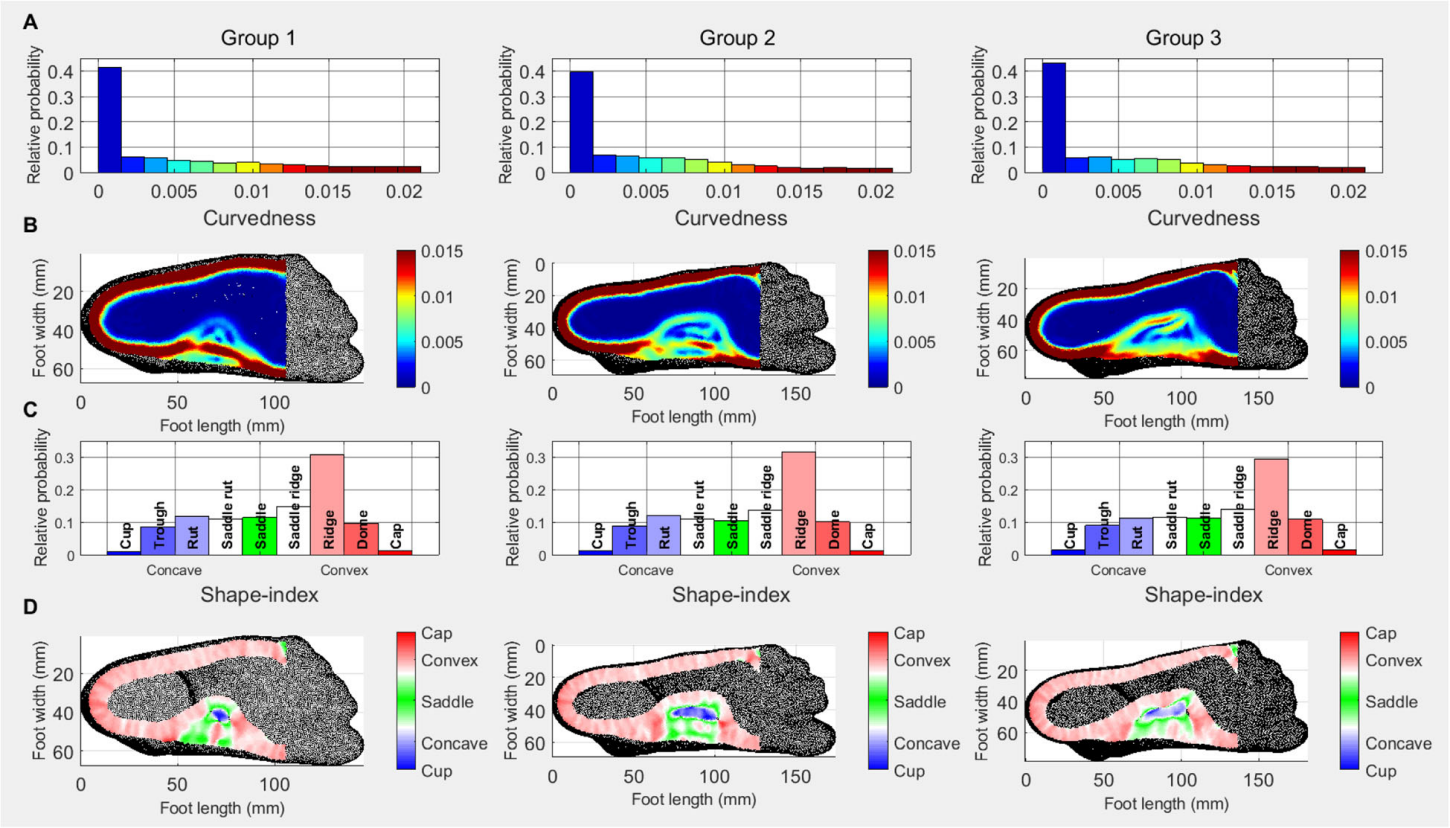
Plantar surface: Relative probability histograms: **a** curvedness **c** shape-index; Heat maps: **b** curvedness **d** shape-index

Due to the plantar surface being mostly flat, the shape descriptor histograms are very different from those of the other surfaces (Fig. 5a). There is a slight increase in the lowest curvedness values in Group 3. The curvedness heat maps (Fig. 5b) show the development of the medial longitudinal arch (MLA) across the age groups. With age, medium curvedness vales extend into the plantar surface from the medial side and create an arch shaped area, incorporating lower curvedness values, and in Groups 2 and 3, some medium values too. The curvedness maps also show that with age the curvedness of the medial edge of the midfoot (red line on the Group 1 participant’s foot) decreases, possibly indicating the development of the MLA.

The shape-index data (Fig. 5c) show a slight decrease in ridge shape in Group 3. The shape-index heat maps (Fig. 5d) are consistent with those of the curvedness and show a development of a convex arch on the medial side extending into the plantar surface, with an increasing area of concavity within it as age increases.

## Discussion

The purpose of this study was to determine whether 3D shape descriptors derived from 3D scanning can identify differences in foot shape in children of three different age groups. The findings from this study demonstrated the ability of 3D shape descriptors to identify and locate differences between age groups and to provide 3D shape information about the development of anatomical structures.

The changes in the distribution of the curvedness data in the dorsal, medial and lateral surfaces indicate that the overall shape of the foot (excluding the plantar surface and the emerging anatomical landmarks) becomes less rounded with age. This is likely to be related to the changing proportions of the foot as it becomes more slender with age (smaller width and circumference measures along with larger length parameters), as suggested in previous studies [11, 13, 14]. These changes are supported by the shape-index histograms of the same surfaces, showing increasing areas of concavity (indented) among the convex (protruding) areas.

Visual inspection of the curvedness heat maps of the dorsal, medial and lateral surfaces demonstrated the emerging bony landmarks. These are highlighted by higher curvedness at the anatomical sites, with flatter regions surrounding them. The shape-index heat maps of the lateral and medial surfaces show that the increase in concavity is also likely to be related to the emergence of bony landmarks creating “valleys” around them. The increase in concave and saddle ridge shapes on the dorsal surface histograms, along with the changes on the shape-index heat maps, suggest similar changes to the medial and lateral surfaces. This include the changing proportions of the foot and an emergence of bony architecture exposing the developing cuneiform bones.

These findings also confirm previous results where younger children’s feet were found to be more robust (larger width and circumference measures along with smaller length measures) based on linear 2D parameters [13]. However, while the circumference and width measures only give us one measure for the whole foot, the heat maps and 3D shape descriptors used in this study can identify specific regions of the foot where the 3D shape changes (e.g. robust to slender) occur.

When considering the plantar surface, the increase in lower curvedness values (see Fig. 4a) suggest an increase in the flat (weight bearing) areas of the plantar surface. The increase in concave areas shown on the shape-index histograms suggests the increase of the area of the MLA. The development of the MLA with age is visible on both the curvedness and shape-index heat maps of the plantar surface. Previous reports have demonstrated increasing area of the MLA during development, however, these studies used 2D foot print measures [7, 31] or contact area [11] and do not allow for the description and the quantification of the surface of the MLA, hence neglecting the 3D properties of a multi-planar structure [12]. A study by Chang, Lin [32] used an indirect measure of the 3D shape of the MLA, the arch volume (the volume under the MLA) and reported an increase with age. In addition to the results of the existing studies, our data using 3D scanning and shape descriptor analysis, has described an increase in concavity of the MLA surface. These data might help us begin to understand more about the structural and functional development of the surrounding soft and bony tissues as a weight bearing, shock absorbing and propulsive unit.

The findings reported in this study demonstrate the potential for greater utilisation of technology and 3D shape descriptors in foot morphology research, as well as in applied environments such as footwear industry and clinical practice. The results reiterate previous recommendations from Telfer and Woodburn [33] which promoted greater utilisation of 3D scanners. This is supported by further work which has explored [34] or recommended [35] the use of 3D shape data in the clinical assessment of the foot shape.

Although there are several current measures which clinicians use to describe foot shape, the relationship between these measures and function is unclear. The measures used in the current study describe shape at a higher resolution with more accuracy and relevance, than current options due to their 3D nature and whole foot approach. We propose that this type of data derived from 3D foot scanning may be able to clarify links between morphology and function. The potential of the approach to provide a clinician with a clear morphological map of the foot and changes between age groups (or between ages within an individual) is an attempt to translate basic research into measures which can be interpreted in an applied clinical setting.

Three-dimensional shape descriptors have shown good potential in isolating changes in foot structure across childhood. Once established, there is the potential that three-dimensional shape data will be beneficial for understanding more about the morphological development of the foot, improving diagnosis techniques, tracking changes over time (for example, pre and post intervention), orthotics and footwear design, understanding disease process and its impact on foot development.

The intention of this work was to demonstrate the potential role of 3D shape descriptors derived from 3D scanning in describing the trajectory of foot shape development and paediatric foot assessment. Although further work is needed to support the translation of the findings into clinical practice, the small sample size enabled a pilot analysis of data, to be used in further research. In this study, 3D shape descriptor heat maps were used, which were based on individual participant foot data, but were checked to be a representative example from that age group. The authors are currently collecting 3D data for a larger cohort of children to further understand the two- and three-dimensional shape development of the paediatric foot. This further work will help to advance understanding of the role of 3D shape descriptors in the quantification of children’s foot shape.

## Conclusions

Previous literature using 3D technology in foot development research has provided a time efficient and accurate way of capturing two dimensional linear foot measures. This study provides further justification for the use of 3D scanning and 3D shape data in foot development research, and it serves as evidence that 3D shape descriptors derived from 3D foot scans can help in identifying, locating and describing changes in foot structure during development. The parameters used in this study can be applied to the evaluation of the foot surface to establish a normative dataset of the paediatric foot in the future. Further work is needed in order to locate changes and variance in 3D shape across childhood with a larger sample size. The data reported in this study can help us understand the 3D development of children’s feet and could have a number of clinical and industry applications.

## Data Availability

The datasets used and/or analysed during the current study are available from the corresponding author on reasonable request.

## Abbreviations

2D: Two dimensional
3D: Three dimensional
MLA: Medial longitudinal arch
Stl: Stereolithography
